# Effects of genetic information on memory for severity of depressive symptoms

**DOI:** 10.1371/journal.pone.0239714

**Published:** 2020-10-14

**Authors:** Woo-kyoung Ahn, Alma Bitran, Matthew Lebowitz

**Affiliations:** 1 Department of Psychology, Yale University, New Haven, Connecticut, United States of America; 2 Department of Psychiatry, Columbia University, New York, New York, United States of America; Semmelweis University, HUNGARY

## Abstract

The general public is increasingly aware of the role of genes in causing depression. Recent studies have begun uncovering unintended negative consequences of learning about a person’s genetic susceptibility to disorders. Because people tend to believe that genes determine one’s identity, having genes related to a disorder can be misinterpreted as equivalent to having the disorder. Consequently, learning that a person is genetically predisposed to depression can make people misremember mild depression as more severe. Participants across three experiments read a target vignette about a character displaying mild depressive symptoms, while descriptions of the character’s genetic susceptibility to depression were experimentally manipulated. Participants then read a foil vignette describing a character with more severe depressive symptoms. Afterwards, participants who had learned that the target character was genetically predisposed to depression were comparatively more likely to misremember the target symptoms as being severe, when in fact they were mild. This pattern of results was obtained among both laypeople (Experiments 1 and 2) and practicing master’s-level, but not doctoral-level, mental health clinicians (Experiment 3). Given that depression is diagnosed primarily based on a person’s memory of depressive symptoms, the current findings suggest that genetic information about depression may lead to over-diagnosis of depression.

## Introduction

Major depression, one of the leading causes of disability in the world [1], is increasingly explained in terms of biological mechanisms. In particular, there has been a rapid growth in our understanding of the genetics of depression. Recently, Howard and colleagues [2] reported a meta-analysis identifying 87 genetic variants significantly associated with depression in the largest ever genome-wide association study for depression with a sample size of over 1 million.

With advances in the genetics of depression, both laypeople and clinicians are frequently exposed to genetic information. Howard and colleagues [2] postulated that clinicians may someday use polygenic risk scores to identify individuals at risk of depression. The general public is also increasingly embracing biogenetic explanations for depression [3, 4].Furthermore, the general public is more and more aware of their own genetic make-up, as personalized genetic testing, such as that offered by 23andMe, is readily available [5]. Thus, it is high time to systematically investigate how attributing depression to DNA affects laypeople’s as well as clinicians’ beliefs and behaviors.

Attributing mental disorders to genes has been shown to have some positive impacts on people’s attitudes towards mental disorders. For instance, since people do not have control over their genes, they may be blamed less for symptoms that are attributed to genetic causes [6]. Genetic information can also be used to offer more individually tailored treatments of disorders [7].

Nonetheless, there are negative consequences as well. For instance, clinicians’ empathy for hypothetical clients was lower when their symptoms were described as being biogenetically, compared to psychosocially, caused [8]. Laypeople in particular appear to over-interpret the importance of genetics, leading genetic explanations to take priority over environmental explanations [9]. Furthermore, genetic explanations can lead laypeople to be pessimistic about the prognoses associated with mental disorders [10, 11] and decrease people’s confidence in their ability to cope with symptoms [12]. Additionally, the more people attribute mental disorders to genetic factors, the more strongly they desire social distance from those with mental disorders [13].

The negative effects of genetic attributions have been explained in terms of genetic essentialism [10, 14]. According to the genetic essentialism account, people falsely believe that a person’s immutable essence is defined by their DNA. Because the essence is immutable and natural, mental disorders that are genetically caused are believed to be more challenging to treat. Furthermore, since the essence defines the person’s identity, those with genetically caused mental disorders would be viewed as fundamentally different from others, increasing the desire to maintain social distance. Indeed, people also think that a mental disorder is a more serious problem when it is genetically caused, not only because it is more persistent but also because it is a problem affecting one’s essence [15].

The current study presents a novel effect of genetic attributions for depression, predicted by genetic essentialism. If genes are believed to determine one’s identity, then learning that a person is genetically predisposed to a certain condition would change how the person is perceived or remembered. For instance, upon learning that Erica is genetically predisposed to major depression, one might misremember or misinterpret Erica’s mildly sad mood to be more severe or pervasive than it actually is, because people assume that Erica is essentially a depressed person.

Such effects could be predicted to occur in much the same way that people have been found to use stereotypes as a lens through which to organize new information about others [16] or to preferentially attend to and remember information that confirms their pre-existing beliefs [17, 18]. In particular, there have been numerous demonstrations of effects of existing beliefs on memory distortion [19]. For instance, after reading a list of words from a common theme (e.g., thread, pin, sharp), participants falsely “remembered” related words (e.g., needle) that were not presented in the list [20]. Kim and Ahn [21] recently showed that people can falsely remember depressive symptoms due to stereotypes they hold about competent people [21]. Laypeople appear to believe that highly competent people would be better at managing depression. As a result, although participants saw identical descriptions of symptoms of depression in vignettes, the competent character’s symptoms were remembered to be less severe than a less competent character’s symptoms. Likewise, because of the kind of genetic essentialist assumptions mentioned above, people may hold the stereotype that genetically caused depression is especially serious or pervasive, and as a result, genetically caused depression may be misremembered as being more severe than it truly was.

Based on this rationale, the current study examines how genetic information can distort one’s memory for symptoms. We predict that people described to have genetic predispositions to depression would be categorized as being depressed people, and as a result, perceptions of their behaviors may be assimilated to fit with such a categorization. Previously, it was shown that leading people to believe they have a genetic predisposition to depression caused them to remember themselves as having been depressed [22]. Yet, this earlier demonstration did not measure the actual levels of participants’ depression, and thus falls short of serving as evidence for memory distorted by genetic information. It also did not examine whether people’s memory for other people’s symptoms can be distorted by genetic information–an issue that would be relevant when considering clinicians’ memory for clients’ symptoms.

To provide direct demonstrations of distorted memory of other people’s depressive symptoms, we used methods previously developed to examine the effects of stereotypes on memory distortion [21]. Participants read a vignette about a target character who was described as exhibiting a mild form of depression. In two of three conditions, the character was described as having taken a genetic test, which revealed that the character was genetically predisposed to depression (gene-present condition) or not (gene-absent condition). In the control condition, there was no mention of genetic testing. After reading about the target, participants read about a foil character who was described as exhibiting more severe depressive symptoms. We reasoned that if participants associated a genetic cause with greater severity, they would be more likely to mistakenly identify the foil character’s (more severe) symptoms as the target character’s symptoms in the gene-present condition than in the gene-absent or control conditions.

Experiment 1 used a female version of the stimuli and Experiment 2 used a male version of the stimuli to confirm the replicability of the findings. While Experiments 1 and 2 tested laypeople, Experiment 3 offers the first empirical investigation of how genetic information affects practicing clinicians’ memory for depressive symptoms.

## Experiment 1

### Materials and methods

The institutional review board of Yale University has approved the current study.

Participants (N = 789) were recruited from Amazon.com’s Mechanical Turk platform in exchange for a small payment. Of these, 40.6% were female; 30.3% were 18–29 years old, and 49.7% were 30–49 years old; 77.9% were white. Measures of income, education, and socioeconomic status were not collected. Study procedures were administered online through Qualtrics.com.

Before beginning the study procedures, participants were provided with an online informed consent form that described basic information about the study. Only those who clicked a check-box labeled, “I have read the above information and agree to participate in the study” could proceed.

Participants were randomly assigned to either the gene-present (N = 262), the gene-absent (N = 264), or the control (N = 263) condition. Participants first read the target vignette in their condition, followed by the foil vignette. Both the foil and target vignettes were presented one sentence at a time, and participants read each at their own pace.

The sentences used for the foil and target vignettes are displayed in Table 1. The target and the foil descriptions each consisted of 9 sentences, 3 of which were about depressive symptoms (sentences 6–8 in Table 1). The target character’s depressive symptoms were identical across the three conditions, and the foil character’s depressive symptoms were more severe forms of those symptoms.

**Table 1:** Stimuli used in Experiments 1 and 3.

Sentence Number	Target			Foil
	Gene-present Condition	Control Condition	Gene-absent Condition	
1	Erica is a 38-year old schoolteacher.	Erica is a 38-year old schoolteacher.	Erica is a 38-year old schoolteacher.	Michelle is a 36-year-old saleswoman.
2	She teaches history to sixth-graders, and has always loved working with kids and teaching.	She teaches history to sixth-graders, and has always loved working with kids and teaching.	She teaches history to sixth-graders, and has always loved working with kids and teaching.	She sells insurance to private clients, and is warm and well-liked among her coworkers.
3	Recently, her doctor recommended that she have genetic testing, so she submitted a saliva sample and waited for a few days.	She is well-liked among her students, and has been happily married for eleven years.	Erica recently received a home DNA test as a holiday gift, so she mailed out her saliva sample and waited for a few days.	She likes to cook meals with her friends and go for walks.
4	The testing revealed that she has a combination of several genes that are linked to major depressive disorder.	Erica recently had a dentist's appointment, where she learned that she needs to schedule a follow-up for a tooth cleaning and a cavity filling.	When the testing results were sent to her, she learned among other things that she does not have any genes associated with major depressive disorder.	She recently went to the DMV to renew her driver's license, which was due to expire within the next few months.
5	The results of the genetic test made sense to her, because Erica's mother suffered from depression before Erica was born.	Her dentist explained that regular flossing is necessary to maintaining proper dental hygiene.	The results of the genetic test made sense to her, because none of her parents or siblings has ever had depression.	Michelle went to college in-state, and lives not too far from where she grew up.
6	**Lately, she has told her best friend that she has been feeling tired and a little “down.”**	**Lately, she has told her best friend that she has been feeling tired and a little “down.”**	**Lately, she has told her best friend that she has been feeling tired and a little “down.”**	**Lately, she said that she is completely exhausted and "very depressed."**
7	**She also noticed that she is a bit less interested in the things she used to enjoy.**	**She also noticed that she is a bit less interested in the things she used to enjoy.**	**She also noticed that she is a bit less interested in the things she used to enjoy.**	**She told a friend that she used to have many favorite activities, but now, she cannot care less about any of them.**
8	**For the past week, she has been sleeping less than she usually does.**	**For the past week, she has been sleeping less than she usually does.**	**For the past week, she has been sleeping less than she usually does.**	**She has been unable to sleep more than four hours a day for the past three weeks.**
9	When Erica saw her uncle recently at a family reunion, he told her that he also had depression earlier in his life.	It's been a cool and moderate past couple of weeks in northern Connecticut, where she lives.	Erica assumed that this is all because she has been stressed out at work recently.	The weather has been average these past few weeks in Massachusetts, where she lives.

In the gene-present and the gene-absent conditions, sentences 4, 5, and 9 conveyed the genetic information and the information intended to corroborate the genetic test results. The family history information (sentences 5 and 9) used in the gene-present condition was carefully created such that it did not imply that Erica was raised by a depressed caregiver (e.g., her mother was depressed before she was born, and the uncle who was depressed was seen at a family reunion). Thus, it was intended to provide family-history information to corroborate the etiological role of genetics uncovered by the genetic test, without also suggesting environmental contributions of the family environment to Erica’s depression.

In addition, sentence 3, which described what prompted Erica to take the genetic testing was different between the gene-present and the gene-absent condition. In the gene-present condition, her doctor recommended the testing, suggesting that the doctor suspected that Erica might be genetically predisposed to depression, further substantiating the positive test results. In the gene-absent condition, however, it was not stated that the doctor ordered the genetic test, because such information could be interpreted as implying that there were reasons to suspect that Erica may be genetically predisposed to depression.

After reading both vignettes, in order to introduce a delay before the memory task, participants saw a series of 20 pictures and identified whether each was a building or a house. Each response had to be made within 2 s to roughly equate the duration of this task across participants.

Afterwards, all participants received a surprise memory task. Participants were told that the purpose of the task was to check what they remembered about the target character (i.e., Erica in this study). They were told that a sentence must contain the exact wording they remember to be rated as true. Then, participants rated each sentence on a scale from 1 (“Definitely True, or did appear”) to 6 (“Definitely False, or did not appear”).

Of the 12 sentences used in the memory task, six were *studied* items (i.e., sentences presented in the target vignette). Four of these were studied items that contained genetically relevant information (denoted S_G_; sentences 3, 4, 5, and 9 in Table 1). Two were studied items that did not contain genetically relevant information (denoted S; sentences 1 and 2 in Table 1). The other six items in the memory task were *lures* (i.e., sentences not presented in the target vignette). Three of the lures were *noncritical* lures (N), which were sentences unrelated to depressive symptoms that were not found anywhere in the actual stimuli (e.g., “Erica has two children”). The other three were *critical* lures (C), which were the more severe depressive symptoms presented in the foil vignette (i.e., sentences 6, 7, and 8 in the foil vignette in Table 1). For all participants, items were presented in the following order: S, N, S, S_G_, C, S_G_, N, S_G_, C, N, S_G_, C. The 3 studied items were presented before the first critical lure to orient the participants to remembering the target character rather than foil character.

### Statistical analysis

Data from all 3 experiments reported in this paper are deposited at https://osf.io/shprj/. Ratings of all critical lures were reverse-coded, such that higher numbers indicate greater confidence that the item was present in the target vignette. These ratings were summed across the 3 critical lures within each participant. Ratings of the noncritical lures were also reverse coded, and we computed a measure of filler-item error by summing all reverse-coded ratings of the noncritical lures and all ratings of the studied items. This filler-item error score was intended to capture participants’ overall level of inaccurate memory for items other than the critical lures. Thus, the filler-error score was entered as a covariate in all our analyses reported in the paper in order to ensure that differences in overall memory would not obscure differences in the critical lure scores. IBM SPSS Statistics Version 26 was used for statistical analyses. For the main analyses, we conducted a one-way ANCOVA with condition as the between-subjects independent variable, critical lure scores as the dependent variable, and filler-item error scores as a covariate.

### Results

**Fig 1:**
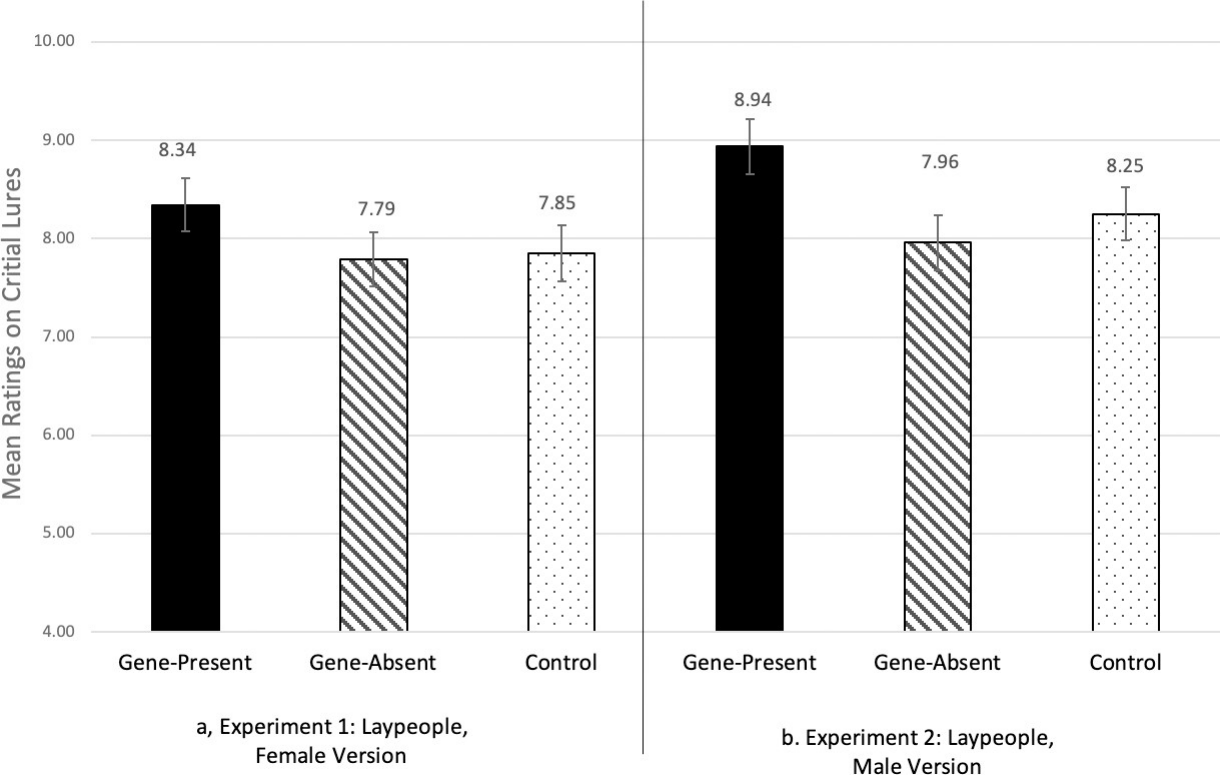
Critical lure scores, by condition, in Experiments 1 and 2. Higher numbers indicate greater confidence that the critical lures were present in the target vignette. Error bars reflect one standard error.

The averages of these critical lure scores, broken down by condition, are shown in panel *a* of Fig 1. The one-way ANCOVA revealed a significant effect of condition, *F*(2,785) = 3.08, *p* = .047, η = .008. We used additional follow-up ANCOVAs, controlling for filler-item error scores, to conduct pairwise comparisons between the conditions. These revealed that participants in the gene-present condition were more confident that critical lures were present in the target vignette (*M* = 8.34, *SD* = 4.41) compared to the control condition (*M* = 7.85, *SD* = 4.57), *F*(1,522) = 4.96, *p* = .026, η_p_ = .009, and also compared to the gene-absent condition (*M* = 7.79, *SD* = 4.48), *F*(1,523) = 3.93, *p* = .048, η_p_ = .007. There was no difference between the control and the gene-absent conditions, *p* = .820.

## Experiment 2

Experiment 2 attempted to replicate the results from Experiment 1 using a male version of the stimuli.

### Materials and methods

The institutional review board of Yale University has approved the current study. The methods were identical to Experiment 1 except for the following. Participants (N = 754) who did not participate in Experiment 1 were recruited for Experiment 2. They were 47.5% female; 29.2% 18–29 years old, and 53.5% 30–49 years old; 76.5% white. Measures of income, education, and socioeconomic status were not collected. The stimuli were identical to those used in Experiment 1 except that the target character’s name was Eric, the foil character’s name was Michael, all pronouns were changed to be male, sentence 5 in the gene-present condition was changed to be about Eric’s father having depression, and sentence 9 in the gene-present condition was changed to be about Eric’s aunt having depression. Participants were randomly assigned to either the gene-present (N = 253), the gene-absent (N = 250), or the control (N = 251) condition.

### Results

Mean critical lure scores, by condition, are shown in panel *b* of Fig 1. A one-way ANCOVA with condition as the between-subjects independent variable, critical lure scores as the dependent variable, and filler-item error scores as a covariate found a significant effect of condition, *F*(2,750) = 3.77, *p* = .023, η_p_ = .010. To conduct pairwise comparisons between the conditions, additional follow-up ANCOVAs, controlling for filler-item error scores, were conducted. Participants in the gene-present condition were more confident that critical lures were present in the target (*M* = 8.94, *SD* = 4.52) compared to the control condition (*M* = 8.25, *SD* = 4.27), *F*(1,501) = 4.31, *p* = .038, η_p_ = .009, and also compared to the gene-absent condition (*M* = 7.96, *SD* = 4.48), *F*(1,500) = 6.32, *p* = .012, η_p_ = .012. There was no difference between the control and the gene-absent conditions, *p* = .70.

## Experiment 3

In the final experiment, we examined whether memory distortions like those observed in Experiments 1 and 2 would be found even among practicing mental health clinicians. As mentioned earlier, clinicians may use genetic information about susceptibility to major depression in clinical settings in the future [2]. In doing so, clinicians may learn of a patient’s genetic predisposition before ever meeting the patient. Thus, it is important to understand whether clinicians’ interpretations of subsequent information that they learn about patients might be biased by genetic information.

There are different types of mental health clinicians with varying licensure requirements, some of which require a master’s degree, while others require a doctoral degree. Experiment 3 recruited clinicians with master’s degrees and clinicians with doctoral-level degrees, to enhance the generalizability of findings and enable finer-grained analyses. For instance, it is possible that those with higher academic degrees may differ more from laypeople in the current task than do those with lower degrees.

### Materials and methods

The institutional review board of Yale University has approved the current study.

Master’s-level clinicians and doctoral-level clinicians participated through separate recruitment streams. To recruit doctoral-level clinicians, we e-mailed 513 clinicians across the U.S. who posted their practice and e-mail address in the American Psychological Association’s psychologist locator website (https://locator.apa.org/landing/). They received a URL for completing the study online, and 190 responded. To recruit master’s-level clinicians, we obtained a list of licensed mental health counselors through the Massachusetts Division of Professional Licensure. Using the mailing addresses provided in this list, 1,200 postcards were sent out with the URL for the study. From this sample, 127 responded. Participants received $5 amazon.com gift cards for their participation.

Of 317 who attempted the survey, the following number of participants were excluded: 28 did not complete the study, 6 did not confirm that they were licensed as a clinician, 4 did not fill out the year in which they obtained their license (the duration of licensure is used as a moderator in the analyses reported in S1 File), and 2 reported their only credential as MD or LCSW (deviating from the recruitment methods which targeted psychologists and mental-health counselors rather than physicians or social workers). The remaining 277 participants’ data were included in analyses. Of these, 113 indicated that they held a Ph.D. only, 109 indicated that they held a master’s degree only, 46 indicated that they held a PsyD, and the remainder indicated that they held an EdD (N = 6), or a combination of some of these credentials (N = 3). The participants were 70.4% female, 86.3% white, and had been licensed for an average of 15.0 years (SD = 13.4, min = 0, max = 50). The procedure, design, and stimuli were identical to those in Experiment 1.

### Statistical analysis

As in Experiments 1 and 2, we used ANCOVA with filler-item error scores as a covariate to analyze the differences among conditions on critical lure scores controlling for overall memory performance. This time, however, given that participants were drawn from two samples, one consisting of psychologists with doctoral degrees and another consisting of master’s-level mental health counselors, we examined whether type of degree—doctorate (n = 168) vs. master’s (n = 109)—interacted with the effect of condition. Thus, the main analysis used a 3 (condition) X 2 (degree) ANCOVA with critical lure scores as the dependent variable and filler-item error scores as a covariate using SPSS.

### Results

**Fig 2:**
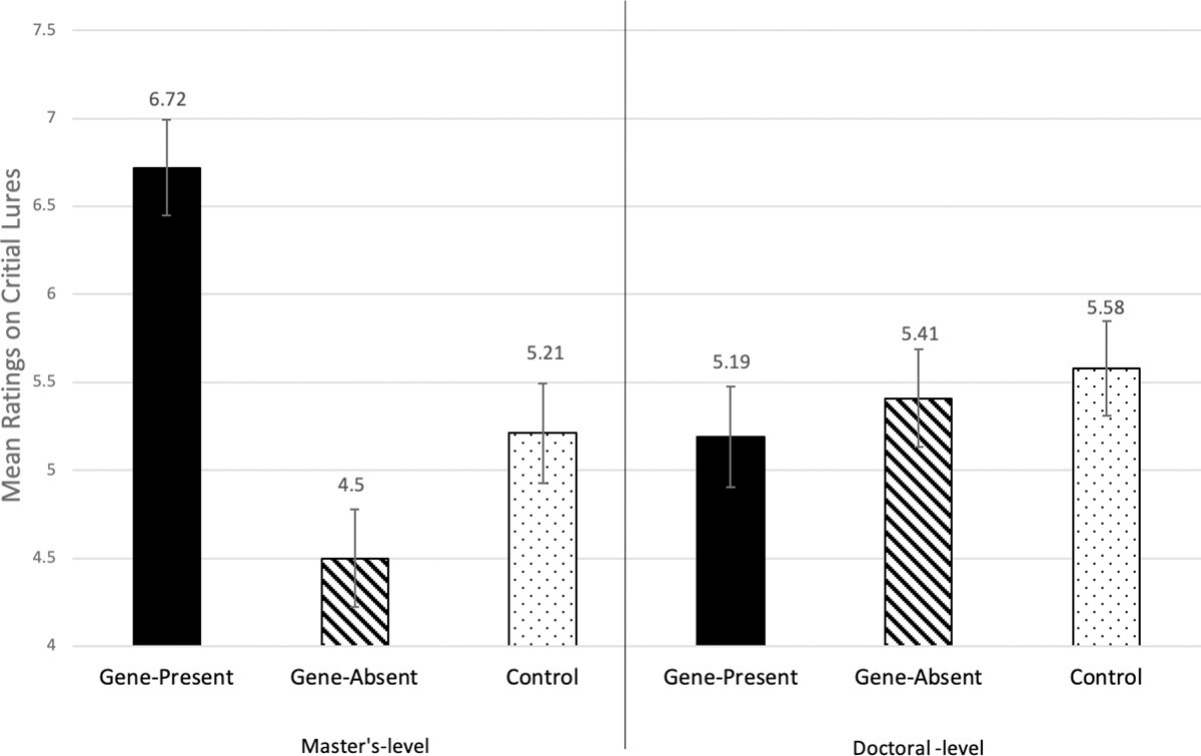
Critical lure scores, by condition, among master’s-level and doctoral-level clinicians in Experiment 3. Higher numbers indicate greater confidence that the critical lures were present in the target vignette. Error bars reflect one standard error.

The 3 (condition) X 2 (degree) ANCOVA found no significant main effect of degree, p = 0.98, but a significant main effect of condition, *F*(2,270) = 4.06, *p* = .018, η_p_ = .029, as well as a marginally significant interaction effect, *F*(2,270) = 2.66, *p* = .072, η_p_ = .019. Although the main effect of condition was significant with the mean critical lure scores in the gene-present condition being the highest as in Experiments 1 and 2, and the interaction effect was only marginally significant using a conventional cutoff, the effect of the gene-present condition appeared to be driven by clinicians with a master’s degree, as illustrated in Fig 2.

Thus, instead of simply concluding that all clinicians are affected by the genetic information, we carried out separate ANCOVAs within each subset of clinicians (i.e., those with master’s degrees and those with doctoral degrees), with condition as the between-subjects independent variable and filler-item error scores as a covariate. For those with a doctoral degree, there was no significant effect of condition, p = .74. For those with a master’s degree, however, there was a significant effect of condition, *F*(2,105) = 6.04, *p* = .003, η_p_ = .103. To conduct pairwise comparisons between the conditions for those with a master’s degree, additional follow-up ANCOVAs, controlling for filler-item error scores, were conducted. Master’s-level clinicians in the gene-present condition were more confident that critical lures were present in the target vignette (*N* = 39, *M* = 6.72, *SD* = 4.18) compared to the control condition (*N* = 34, *M* = 5.21, *SD* = 3.04), *F*(1,70) = 9.44, *p* = .003, η_p_ = .119, and also compared to the gene-absent condition (*N* = 36, *M* = 4.50, *SD* = 2.06), *F*(1,72) = 8.02, *p* = .006, η_p_ = .10. There was no difference between the control and the gene-absent conditions, *p* = .38.

To summarize, in Experiment 3 the effect of genetic information on memory distortions that was observed among lay participants in Experiments 1 and 2 was replicated among clinicians with a master’s degree but not among those with a doctoral degree. This could suggest that their higher level of training (i.e., greater expertise) protected doctoral-level clinicians from the kinds of memory distortions observed among laypeople and master’s-level clinicians. (See S1 File for additional analyses with the duration of licensure as a moderator.)

## Discussion

### Summary of results

Across three experiments, participants who read a target vignette about a character displaying mild depressive symptoms were more likely to misremember that target character as having experienced the more severe symptoms detailed in a foil vignette if the target character was described as having been genetically predisposed to depression than not. We verified that this memory distortion was specific to the participants’ recollection of depressive symptoms by controlling for overall levels of memory inaccuracy using a variable that represented memory inaccuracies for stimuli other than the depressive symptoms. These results were replicated with both female and male versions of the characters who displayed depressive symptoms. The results were also replicated not only with laypeople but also with practicing clinicians with master’s-level (as opposed to doctoral) training.

These findings are consistent with a recent study in which participants who were experimentally led to believe that they were genetically predisposed to depression reported having had a higher level of depressive symptoms in the past two weeks compared to participants who were not [22]. Given that these participants in the earlier study were randomly assigned to one of the experimental conditions, the genetic feedback was most likely to have caused the elevated recall of depressive symptoms. The current results showed more direct evidence of memory distorted by genetic information. Even when they were explicitly told that the target character had only a mild level of depression, genetic information caused participants to be more confident that the target character had a more severe level of depression.

### Implications of findings

The current findings have significant clinical as well as public health implications, as personalized genetic information is likely to become more available for clinical use in mental health [23]. Most mental disorders are diagnosed based on patients’ self-reports; the diagnostic process relies on a patient’s memory of having experienced symptoms and a clinician’s subjective understanding of that self-report. Thus, genetic information about depression may lead to over-diagnosis of depression, if symptoms are misremembered as being more severe than they actually are. Our results show that clinicians and patients should be aware of potential memory biases that could be elicited by genetic information.

The present results also have significant implications for the stigmatization of mental disorders, as they present the first empirical demonstration that genetic information about other people can alter the perceiver’s memory for other people’s depressive symptoms. When one learns that another person has a genetic predisposition to depression, any depressive experiences that the person has, even those in the subclinical range, may be interpreted or remembered as being more severe. Given that psychopathology is strongly stigmatized [9], biases that lead people to be perceived as having more severe symptoms are also likely to lead to more stigmatization.

Experiment 3 also found an effect of genetic information on memory for depressive symptoms among mental health counselors with a master’s degree, but not among clinicians with more advanced degrees. Perhaps clinicians with more advanced degrees have more accurate knowledge about genetics, preventing their memories from becoming biased by genetic information. Future studies should consider examining whether genetic background knowledge moderates the effects reported here among clinicians as well as laypeople. Such findings would be informative in devising intervention strategies.

### Limitations

Although the effect of genetic information was replicated across three experiments, the effect size tended to be small except among the master’s-level clinicians. However, it is possible that similar effects could be larger in real-world contexts, for a number of reasons. In the current experiments, the delay between the presentation of the target symptoms and the memory task was only about 2 minutes for most participants, and with a longer delay, memory distortions could become more pronounced. In addition, while participants read the depressive symptoms all at once in the current experiments, people in real-life situations are more likely to learn about others’ symptoms in a sequential and scattered manner, which could lead to greater memory distortions. This is because once genetic information engenders the belief that a person is more seriously depressed than they actually are, this belief can in turn exacerbate the biased assimilation of subsequent information. Finally, in the current study the depressive symptoms were explicitly described to be not severe, but in real-life contexts the signs of depressive symptoms would likely be much more ambiguous and complex. Just as stereotype-based information processing is more pronounced when the input is more complex [16], the effect of genetic information may be more pronounced in complex, real-life situations.

A related issue is that perhaps the experimental manipulation was too strong. As discussed earlier, in the gene-absent and the gene-present condition, we introduced information about family history that was intended to corroborate the genetic test results, as well as information about what prompted the character in the vignette to take a genetic test. Although the wording of the family history information was carefully chosen so as to avoid suggesting environmental causes for the character’s depression (e.g., the mother’s depression occurred before the main character was born), it is possible that participants might have assumed that the mother’s history of depression influenced the target character’s mental health through non-genetic means. Additionally, while the fact that a doctor recommended genetic testing, which appeared only in the gene-present condition, was intended to further substantiate the positive genetic test results, participants might have misinterpreted it as a sign that the main character had more severe depression. Future research can further investigate the robustness of the current findings by using subtler experimental manipulations.

Another limitation of the current study is that only depression was used in the stimuli, so the scope of the effect is unknown. Memory distortions caused by genetic information might be weaker for disorders that are not strongly believed to be biologically based (e.g., personality disorders) [24]. At the same time, the memory distortions may be larger for disorders with greater heritability than major depression (e.g., bipolar disorder). It would be important to examine the effect of genetic feedback across a variety of disorders.

### Conclusion

In conclusion, the present study demonstrated a bias toward perceiving depressive symptoms as being more severe when the person displaying the symptoms has a genetic predisposition to depression. This bias was found among laypeople as well as master’s-level practicing clinicians. These results add to the growing literature showing unintended negative impacts of genetic information. Given the current findings’ implications for potential stigmatization and for the possibility that genetic information could interfere with the accurate diagnosis of depressive disorders, future studies should investigate how such memory distortions can be prevented.

## Supporting information

S1 File (DOCX)
