# Supplementary material for: Effects of genetic information on memory for severity of depressive symptoms
Source: PLoS One. 2020 Oct 14;15(10):e0239714. doi: 10.1371/journal.pone.0239714 (PMC7556482; doi:10.1371/journal.pone.0239714)
Supplement: S1 File — (DOCX) [file pone.0239714.s001.docx]

Supplementary Analyses for Experiment 3

In exploring the potential effect of expertise, another moderator to consider would be the duration of licensure. This variable was confounded with degree; those with a doctoral degree had a longer duration of licensure (*M* = 19.11 years, *SD* = 14.30) than those with a master’s degree (*M* = 8.71 years, *SD* = 8.83), *t*(275) = 6.79, *p*<.001. As such, we did not enter the duration of licensure as another variable when considering the effect of degree. Instead, we examined duration of licensure as a moderator separately, using PROCESS version 3.4 for SPSS (model 1), entering condition as the independent variable, duration of licensure as the moderator, critical lure scores as the dependent variable, and filler-item error scores as a covariate. This analysis included all clinicians from both samples (i.e., both doctoral and master’s-level). The gene-present condition was treated as the reference group; dummy codes were created for the other two conditions. There were significant main effects indicating that, compared to the gene-present condition, critical lure scores were significantly lower in the control condition (b = -2.80, SE = .71, t = -3.95, p = .001) and marginally lower in the gene-absent condition (b = -1.15, SE = .68, t = -1.68, p = .09). The dummy code for the control condition (vs. the genes-present condition) also significantly interacted with duration of licensure (b = .10, SE = .03, t = 2.99, p = .003). This interaction was probed by examining conditional effects of the two dummy codes (for the control condition and the gene-absent conditions, respectively, vs. the genes-present condition) at values of the duration-of-licensure variable representing the 16th percentile (2 years), the 50th percentile (11 years), and the 84th percentile (31 years). For a licensure duration of 2 years, critical lure scores were significantly lower (compared to the gene-present condition) in the control condition (b = -2.60, SE = .66, t = -3.94, p < .001) and marginally lower in the gene-absent condition (b = -1.09, SE = .63, t = -1.72, p = .086). Similarly, for a licensure duration of 11 years, critical lure scores were significantly lower (compared to the gene-present condition) in the control condition (b = -1.67, SE = .50, t = -3.37, p < .001) and marginally lower in the gene-absent condition (b = -.83, SE = .48, t = -1.75, p = .082). However, for a licensure duration of 31 years, neither the control condition nor the gene-absent condition was significantly different from the gene-present condition (*p’*s > .58).

To summarize, these analyses suggested that clinicians with fewer years of experience were more susceptible to this effect of genetic information, whereas those with more years of experience showed no evidence of such memory distortion. This pattern of results might have been obtained because more experienced clinicians have more accurate memory for symptoms, making them less susceptible to the effect of genetic information. Yet, this account is unlikely given previous studies showing that more experienced clinicians’ memory for clients was worse than intermediate-level clinicians (Marsh & Ahn, 2012; see also Garb, 2005). Since those who were less experienced in the current study tended to be the ones without doctoral-level training, it may be the level of training that led to this effect, although future research should attempt to dissociate the two factors.
